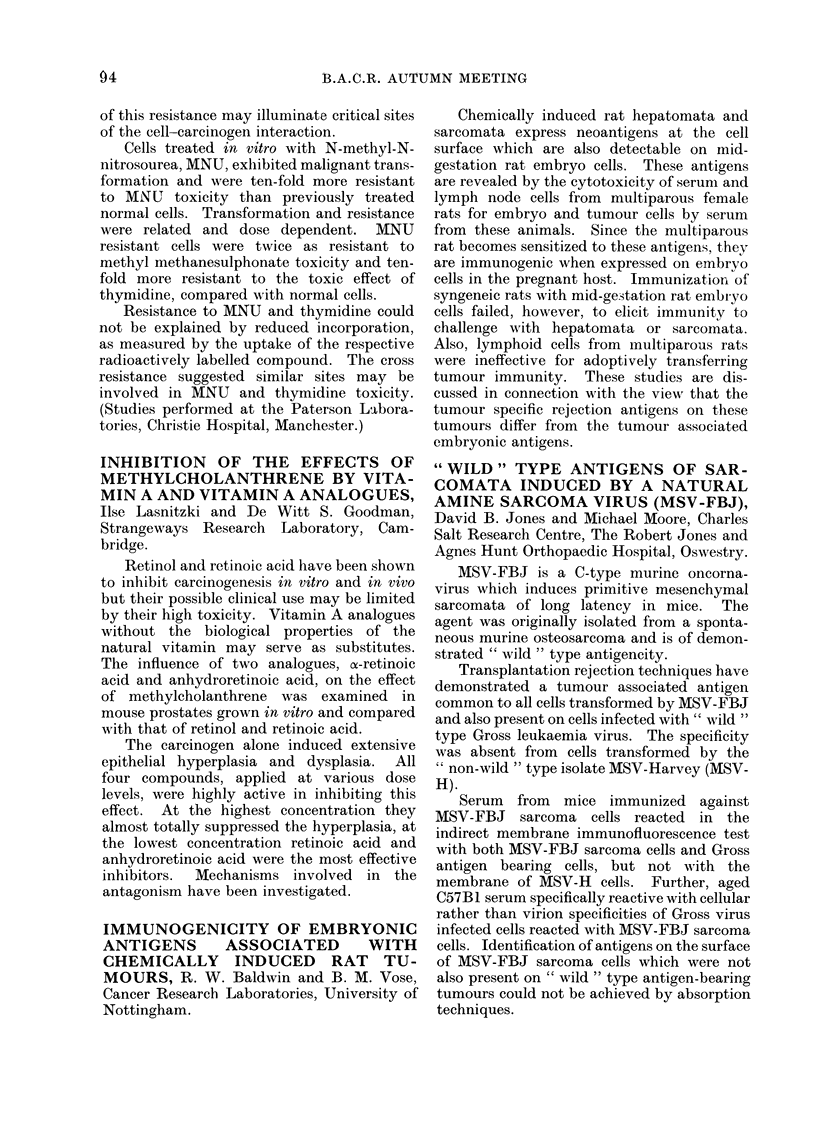# Proceedings: Immunogenicity of embryonic antigens associated with chemically induced rat tumours.

**DOI:** 10.1038/bjc.1974.21

**Published:** 1974-01

**Authors:** R. W. Baldwin, B. M. Vose


**IMMUNOGENICITY OF EMBRYONIC ANTIGENS ASSOCIATED WITH CHEMICALLY INDUCED RAT TUMOURS,** R. W. Baldwin and B. M. Vose, Cancer Research Laboratories, University of Nottingham.

Chemically induced rat hepatomata and sarcomata express neoantigens at the cell surface which are also detectable on mid-gestation rat embryo cells. These antigens are revealed by the cytotoxicity of serum and lymph node cells from multiparous female rats for embryo and tumour cells by serum from these animals. Since the multiparous rat becomes sensitized to these antigens, they are immunogenic when expressed on embryo cells in the pregnant host. Immunization of syngeneic rats with mid-gestation rat embryo cells failed, however, to elicit immunity to challenge with hepatomata or sarcomata. Also, lymphoid cells from multiparous rats were ineffective for adoptively transferring tumour immunity. These studies are discussed in connection with the view that the tumour specific rejection antigens on these tumours differ from the tumour associated embryonic antigens.